# Thickening Supercritical CO_2_ with π-Stacked Co-Polymers: Molecular Insights into the Role of Intermolecular Interaction

**DOI:** 10.3390/polym10030268

**Published:** 2018-03-06

**Authors:** Wenchao Sun, Baojiang Sun, Ying Li, Xiaonan Huang, Haiming Fan, Xinxin Zhao, Haoyang Sun, Wenxia Sun

**Affiliations:** 1 State Key Laboratory of Heavy Oil Processing, China University of Petroleum (East China), Qingdao 266580, China; sunwenchao8306@163.com (W.S.); haimingfan@126.com (H.F.); zhaox@upc.edu.cn (X.Z.); 2 Key Laboratory of Colloid and Interface Chemistry of State Education of Ministry, Shandong University, Jinan 250100, China; yingli@sdu.edu.cn (Y.L.); haoyangsunhaoyang@163.com (H.S.); 3 Department of Chemistry, Capital Normal University, Beijing 100048, China; huangxn@cnu.edu.cn; 4 Geological Logging Company, Shengli Petroleum Engineering Company, Petroleum Engineering Services Limited Company of China Petrochemical Corporation, Dongying 257100, China; sunwenxia0929@sina.com

**Keywords:** supercritical CO_2_, shale gas, thickening agent, co-polymer, molecular simulation

## Abstract

Vinyl Benzoate/Heptadecafluorodecyl acrylate (VBe/HFDA) co-polymers were synthesized and characterized as thickening agents for supercritical carbon dioxide (SC-CO_2_). The solubility and thickening capability of the co-polymer samples in SC-CO_2_ were evaluated by measuring cloud point pressure and relative viscosity. The molecular dynamics (MD) simulation for all atoms was employed to simulate the microscopic molecular behavior and the intermolecular interaction of co-polymer–CO_2_ systems. We found that the introduction of VBe group decreased the polymer–CO_2_ interaction and increased the polymer–polymer interaction, leading to a reduction in solubility of the co-polymers in SC-CO_2_. However, the co-polymer could generate more effective inter-chain interaction and generate more viscosity enhancement compared to the Poly(Heptadecafluorodecyl) (PHFDA) homopolymer due to the driving force provided by π-π stacking of the VBe groups. The optimum molar ratio value for VBe in co-polymers for the viscosity enhancement of SC-CO_2_ was found to be 0.33 in this work. The P(HFDA_0.67_-*co*-VBe_0.33_) was able to enhance the viscosity of SC-CO_2_ by 438 times at 5 wt. %. Less VBe content would result in a lack of intermolecular interaction, although excessive VBe content would generate more intramolecular π-π stacking and less intermolecular π-π stacking. Both conditions reduce the thickening capability of the P(HFDA-*co*-VBe) co-polymer. This work presented the relationship between structure and performance of the co-polymers in SC-CO_2_ by combining experiment and molecular simulations.

## 1. Introduction

Supercritical carbon dioxide (SC-CO_2_) has been applied in many industrial processes due to its good diffusion, heat transfer and mass transfer properties. In unconventional oil and gas exploitation, CO_2_ has been recognized as clean fracturing fluid, which has strong width generation capability [1,2]. SC-CO_2_ does not damage the reservoir and possesses obvious technical advantages for the stimulation of low permeability and water-sensitive reservoir compared to hydraulic fracturing. In addition, CO_2_ is easier to flow back and could replace the natural gas adsorbed in the reservoir [3,4]. The practices of the United States and Canada showed that the stimulation effect of CO_2_ fracturing was especially excellent for shale gas reservoirs [5]. However, the low viscosity of SC-CO_2_ has limited its capability of exporting the proppants into the reservoir fractures to support the oil–gas pathway [6]. Therefore, thickening SC-CO_2_ is essential in improving the technology for the sand fracturing of SC-CO_2_ [7]. The viscosity enhancement of CO_2_ could improve its proppant exporting performance and reduce its filtration into the reservoir [4].

Due to the low solubility of polymer in CO_2_, thickening CO_2_ using polymers was very difficult. The good solubility of polymer in SC-CO_2_ is reflected by the CO_2_-philic group, low surface tension [8] and low glass transition temperature (*T_g_*) [9,10] generally. Tapriyal et al. found that the charge deviation and quadrupole moment allows CO_2_ to play the roles of both electron-accepting Lewis acid and electron-donating Lewis base, which provides it with the ability to interact with specific groups (such as fluorine, carbonyl, carboxyl, etc.) [11]. For example, the hydrogen bond and electron donor–acceptor interaction (EDA) between CO_2_ and carbonyl group was believed to improve the solubility of materials in SC-CO_2_ [12,13]. Wallen et al. demonstrated that, except for the Lewis Acid–Lewis Base (LA–LB) interaction between the carbon of CO_2_ and oxygen of carbonyl in the CO_2_-carbonyl compound systems, there is a cooperative C–H···O interaction which is worth considering in the design of CO_2_-philic materials [14,15,16]. The CO_2_-soluble polymers were generally recognized as containing CO_2_-philic groups with high free volume, low solubility parameters and low glass transition temperatures [17,18,19,20]. 

Beckman et al. put forward the argument that specialized thickeners should be synthesized rather than testing a huge number of commercial polymers [21]. Researchers have explored methods to enhance the CO_2_-philicity by modifying polymers with specific groups [22,23,24]. Due to the strong LA–LB interaction between F and C of CO_2_, fluoropolymer has high solubility in CO_2_, but does not enhance the viscosity of CO_2_ significantly, which may due to the weak polymer–polymer interaction. It was believed that the co-polymer obtained from the combination of a CO_2_-philic monomer and CO_2_-phobic monomer could generate inter-chain associations to thicken CO_2_ by hydrogen bonds, solvo–phobic or van der Waals interactions [25]. It may be an effective strategy to modify the CO_2_-philic polymer by introducing a CO_2_-phobic group in the molecular design of thickening agents for SC-CO_2_ [26,27,28]_._ Enick et al. have synthesized P(HFDA-*co*-St) co-polymers as thickening agents and the experimental results showed that the co-polymers could thicken CO_2_ effectively [4,6]. 

The exploitation of new functional polymer for thickening CO_2_ requires clear knowledge of the effect of molecular structure on performance. It is difficult to obtain the correction by experiments due to the huge cost of time and economy. Molecular simulation has been used to reveal microscopic structure and intermolecular interactions of the polymer–CO_2_ system. Johnson et al. have calculated the interaction between CO_2_ and polymer repeat units by ab initio calculation, but the calculation results were inconsistent with the experimental results. The reason is that the model does not consider the influences of temperature and polymer–polymer interactions [29,30]. Compared to the ab initio calculation, the molecular dynamics simulation (MD) for all atoms is more suitable for the polymer–CO_2_ system and the results are more credible. Liu and Sun have explored the correlation of co-polymer structure with its solubility by MD simulations and obtained good results [31,32,33,34]. The microscopic information of the polymer–CO_2_ system could be used to provide guidance for the exploitation of polymer thickeners. To date, little research about the thickening capability of polymer in SC-CO_2_ simulated by the all-atom MD has been reported as far as we know.

This paper aims to provide insights into the role of intermolecular interactions in the solubility and thickening capability of π-stacked co-polymers by experiments and molecular simulation. Four co-polymers with different compositions were synthesized and characterized. The solubility and thickening capability of the co-polymers in SC-CO_2_ were evaluated by cloud point pressure and relative viscosity measurement experiments. By investigating the co-polymer–CO_2_ interaction energy, equilibrium conformations and radial distribution functions (RDFs), microscopic structure and intermolecular interaction of co-polymer–CO_2_ systems were explored. The relationships of the co-polymer structure and composition with the solubility and thickening capability were revealed.

## 2. Methodology

### 2.1. Synthesis and Characterization

#### 2.1.1. Materials

The materials used in this work were vinyl benzoate (VBe, 99.5%, Alfa Aesar, Heysham, UK), 3,3,4,4,5,5,6,6,7,7,8,8,9,9,10,10,10-heptadecafluorodecyl acrylate (HFDA, 98%, Alfa Aesar, Heysham, UK), 2,2′-Azobisisobutyronitrile (AIBN, 99%, Aladdin Industrial Corporation, City of Industry, CA, USA), 1,1,2-Trichlorotrifluoroethane (99.5%, Aladdin Industrial Corporation, City of Industry, CA, USA), Methanol (99.9%, Aladdin Industrial Corporation, City of Industry, CA, USA) and Chloroform-d (99.8%, Alfa Aesar, Heysham, UK). AIBN was recrystallized twice before use.

#### 2.1.2. Preparation of Co-Polymer

**Scheme 1:**
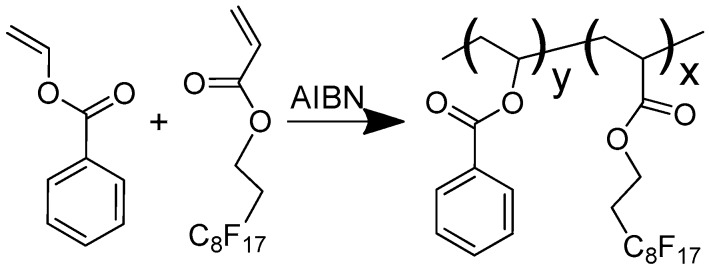
Synthetic procedure of the P(HFDA-*co*-VBe) co-polymer, where *x*: the molar ratio of HFDA and *y*: the molar ratio of VBe.

The Vinyl benzoate/Heptadecafluorodecyl acrylate (VBe/HFDA) co-polymer was synthesized with AIBN as the initiator according to the procedure shown in Scheme 1 [35,36,37]. Two monomers were mixed together in a glass ampoule. AIBN (1.0 mol % relative to monomers) was added as a free radical initiator. The ampoule was sealed under reduced pressure and the polymerization was carried out at 60 °C for 24 h after the oxygen was removed. The product was dissolved in 1,1,2-trichlorotrifluoroethane and then purified twice in methanol. After this, the product collected and dried under vacuum. The co-polymers synthesized were named P(HFDA*_x_*-*co*-VBe*_y_*), ‘*x*’ is the molar ratio of HFDA and *y* is the molar ratio of Vinyl Benzoate. 

#### 2.1.3. Nuclear Magnetic Resonance (NMR) Spectroscopy

^1^H-NMR spectrum of the co-polymer was recorded by Bruker 400 MHz Nuclear Magnetic Resonance Spectrometer (Bruker, Karlsruhe, Germany) with 1,1,2-tritriuorotrichloroethane and Chloroform-d used as the solvent at 20 °C. The spectra were used to analyze the co-polymerization ratio. The co-polymerization ratio of the two monomers was obtained by the integral relative intensity of the chemical shifts of the H atoms belonging to different characteristic proton groups [32,33,34,35,36]. 

#### 2.1.4. Differential Scanning Calorimetry

To evaluate the chain flexibility, glass transition temperature of the co-polymers as measured using the Mettler-Toledo Differential Scanning Calorimeter (Mettler Toledo, Zurich, Switzerland) under the N_2_ atmosphere. The heating rate and the cooling rate were 10 °C/min [22,23,24]. Each sample was measured three times, taking the average of the last two as the result. 

#### 2.1.5. Molar Mass Analysis

Six solutions with different concentrations of the co-polymer sample was dissolved in 1,1,2-trichlorotrifluoroethane, before being filtered through a 0.8-μm filter. The absolute refractive index increments of co-polymer sample, *d_n_*/*d_c_*, were determined by the Optilab rEX extended differential refractive index detector, while the molar mass of the co-polymer sample was measured by Static Light Scattering (SLS) with Wyatt DAWN HELEOS Multi-Angle Laser Light Scatter (Wyatt Technology, Santa Barbara, CA, USA). The light scattering data was measured through a fixed 18-angle detector display simultaneously [38,39,40,41,42,43,44].

#### 2.1.6. Surface Tension Measurements

The surface tension of the co-polymers was determined to evaluate the polymer–polymer interactions. The solution of polymer with 1,1,2-trifluorotriochloroethane was spin-coated on a glass substrate at a rotational speed of 2000 rpm. A homogeneous polymer film was obtained after drying. The contact angles of water–polymer and hexadecane–polymer were measured by DSAHT Contact Angle Meter (KRÜSS GmbH, Hamburg, Germany). After this, the surface tension of the co-polymers was calculated by the two-liquid method to evaluate the polymer–polymer interaction [36,45,46,47].

### 2.2. Cloud Point and Viscosity Measurements

**Scheme 2:**
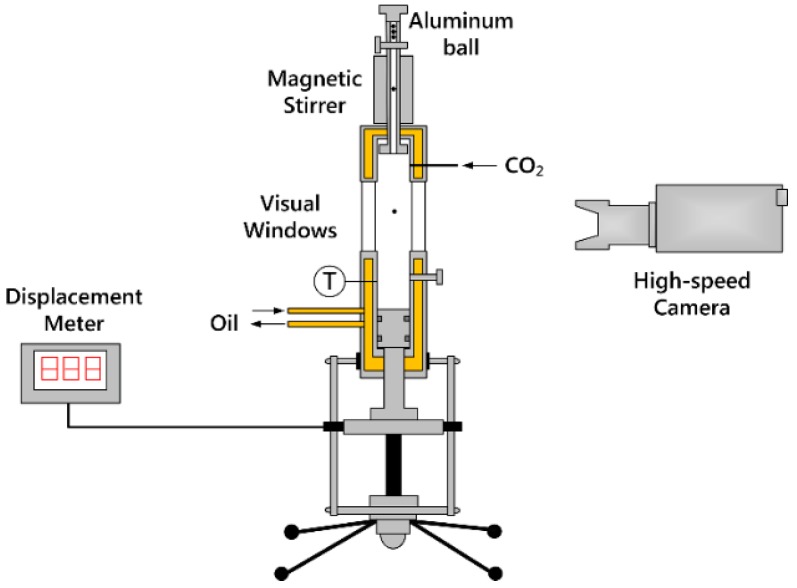
High-temperature and high-pressure visual unit used for cloud point and viscosity measurements.

The core part used for cloud point and viscosity measurements was the high temperature and high pressure visual unit, which is shown in Scheme 2. The visual windows were made of quartz glass and the supporting pressure is 30 MPa. The temperature was controlled by oil bath. The volume of the unit could be changed by moving the piston, which had a moving distance that was determined by displacement meter.

**Figure 1:**
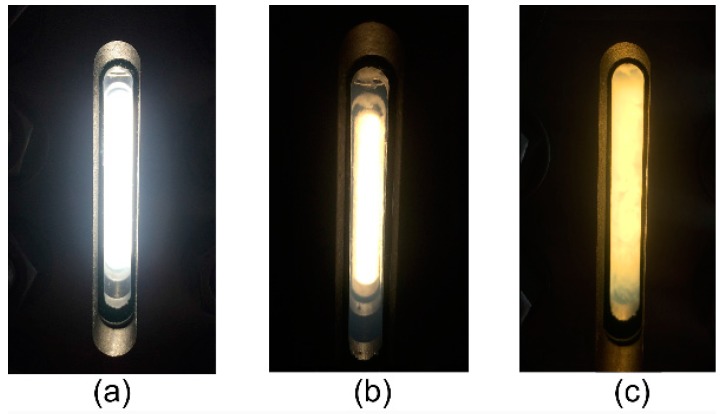
The phase transitions of supercritical CO_2_–polymer system under constant temperature, (**a**) single phase (above cloud point); (**b**) the appearance of the mist (cloud point); and (**c**) two phase (below cloud point)**.**

The polymer–CO_2_ mixture was stirred for 30 min to form a homogeneous system after being heated to the desired temperature, before the piston was moved to bring the system to the desired pressure. The polymer–CO_2_ system was a single phase under the initial conditions after having achieved phase equilibrium. After this, the piston was moved slowly to increase the volume of the unit under constant temperature, before decreasing the pressure until mist appeared. The pressure at which the mist appeared exactly was the cloud point pressure [48,49]. The phase transitions of the CO_2_–polymer system under a constant temperature were shown in Figure 1. The cloud point pressure was determined by repeating three measurements.

The pitching device at the top of the device could put aluminum ball into the visual unit. At the pressures above the cloud point pressure, the process of aluminum ball passing through the visual windows was captured by the high-speed camera at 2000 frames per second and used for the viscosity calculation. The viscosity of CO_2_ and polymer–CO_2_ mixture were calculated by Equation (1) under the pressures above the cloud point pressure [50,51]:

(1) $$\eta =\frac{t(1-\rho_{l}/\rho_{s})}{A\left[1+2\alpha (T-T_{r})\right]\left[1-2\beta (P-P_{r})\right]},$$

where *α* and *β* are the thermal expansion coefficient and the compression coefficient of the visual unit; *T_r_* and *P_r_* are the reference temperature and pressure; *ρ_l_* and *ρ_s_* are the density of the solution and the ball respectively; *t* is the settling time; and *A* is the apparatus constant.

The ratio of the CO_2_–polymer viscosity to the CO_2_ viscosity at the same temperature and pressure, which is also known as the relative viscosity was used to evaluate the viscosification of the co-polymer [21]. The relative viscosity was obtained by Equation (2):

(2) $$\mathrm{Relative}\;\mathrm{viscosity}=\eta_{\mathrm{mix}}/\eta_{{\mathrm{CO}}_{2}},$$

where *η*_mix_ is the viscosity of the CO_2_–polymer mixture and $\eta_{{\mathrm{CO}}_{2}}$ is the viscosity of CO_2_. 

### 2.3. Molecular Dynamics Simulation

**Scheme 3:**
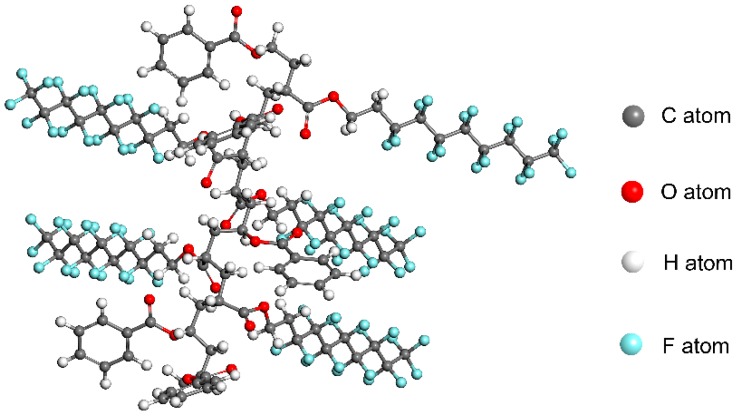
The molecular modeling of P(HFDA-*co*-VBe) co-polymer.

Two types of systems, which are namely the system with eight chains of co-polymer as well as the co-polymer–CO_2_ system with eight chains of co-polymer and 2000 molecules of CO_2_, were simulated by the Material Studio 7.0 (MS) package. All the force-field parameters of CO_2_ and polymers were determined by the COMPASS force-field. Firstly, HFDA and VBe repeat units were established. Four molecular modelings of P(HFDA-*co*-VBe) random co-polymers were constructed with different molar ratios of HFDA to VBe. PHFDA homopolymer was constructed with HFDA repeat units. The molecular modeling of P(HFDA-*co*-VBe) co-polymer is shown in Scheme 3.

Then, CO_2_ and polymer molecules were optimized by the Smart Minimizer of the Discover module. After this, the systems were constructed by the Amorphous Cell module. This module makes the boxes have periodic boundary conditions and constructs quasi-infinite system to represent the macro system precisely. The different systems simulated are shown in Table 1.

**Figure 2:**
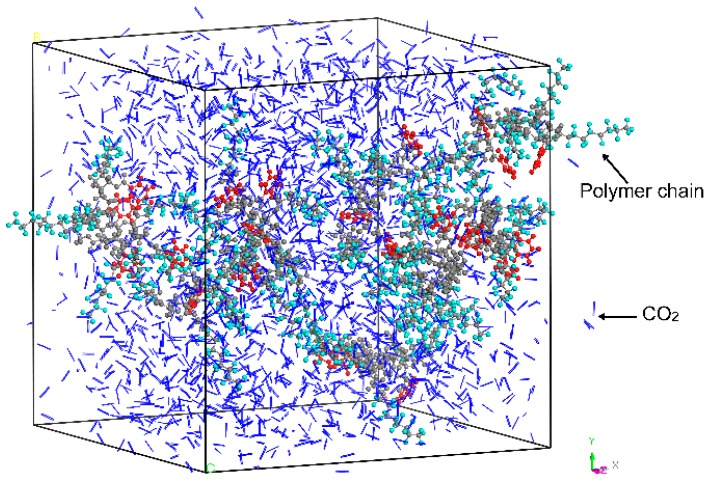
Optimized conformation of the polymer–CO_2_ system (CO_2_ was marked as blue line molecular; while polymer chain was marked as ball and stick figure).

Five cycles of annealing calculation from 300 to 500 K was calculated by Anneal in the Forcite module to delay the systems. The optimized conformation of co-polymer–CO_2_ system is shown in Figure 2.

The MD simulation with 2000 ps was carried out in the NPT (constant-pressure, constant-temperature) ensemble at 25 MPa and 308.2 K. The electrostatic interactions were calculated using the Ewald summing algorithm and the van der Waals interactions were calculated using the atom-based summation. The time step was 1 fs and the frame output interval was 5000 ps. Andersen method was used for the temperature control and Berendsen method was used for the pressure control [33,34]. The final 500 ps equilibrium conformations were used for data analysis.

## 3. Results and Discussion

### 3.1. Synthesis and Characterization

**Figure 3:**
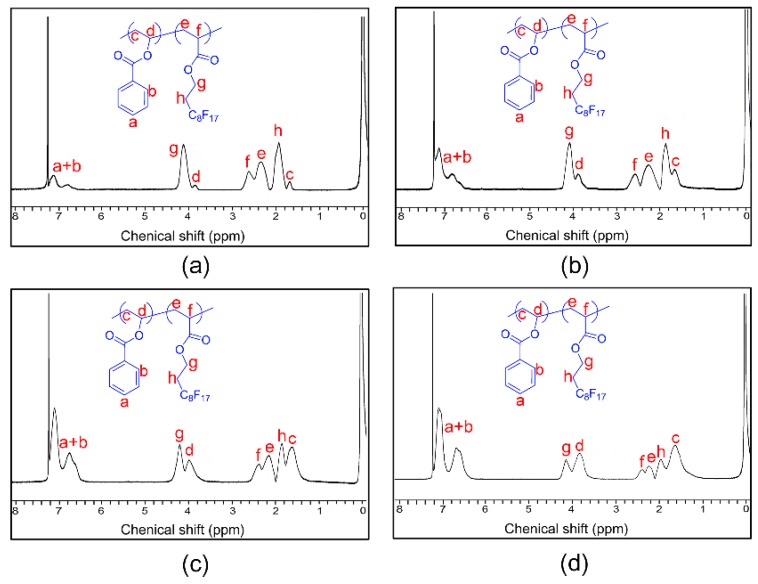
^1^H-NMR spectrums for P(HFDA-*co*-VBe) co-polymers. (**a**) P(HFDA_0.87_-*co*-VBe_0.13_); (**b**) P(HFDA_0.67_-*co*-VBe_0.33_); (**c**) P(HFDA_0.50_-*co*-VBe_0.50_); (**d**) P(HFDA_0.29_-*co*-VBe_0.71_).

The experimental results of the synthesis and physical properties of the co-polymer samples are shown in Table 2. With the enhancement of the molar ratio of VBe, the surface tension and glass transition temperature increased gradually. The molar mass measurement results showed that the differences of molar mass between the co-polymer samples were small, so the effects of molar mass on cloud point pressure and viscosification were not considered in this paper. The co-polymer compositions were determined by comparing the peak intensities of phenyl protons at 6.8 and 7.1 ppm (*a* and *b*) and methylene protons at 1.9 ppm (*h*). Taking the spectrum of P(HFDA_0.87_-*co*-VBe_0.13_) shown in Figure 3a as an example, the chemical shift of the H atoms in HFDA group at the point *h* was in the range of 1.8–2.0 ppm, while the chemical shift of the H atoms in VBe group at the point *a* and *b* was in the range of 6.5–7.2 ppm. The integral relative intensity of the peaks of *h* to *a + b*, was equal to the molar ratio of the two groups, which was *HFDA*/*VBe* = *x*:*y* = *h*/2:(*a + b*)/5 = 0.87:0.13 [32,33,34,35,36]. The co-polymerization ratios of the other co-polymers were obtained by the same method, which is shown in Figure 3b–d.

### 3.2. Solubility of Co-Polymers in SC-CO_2_

**Figure 4:**
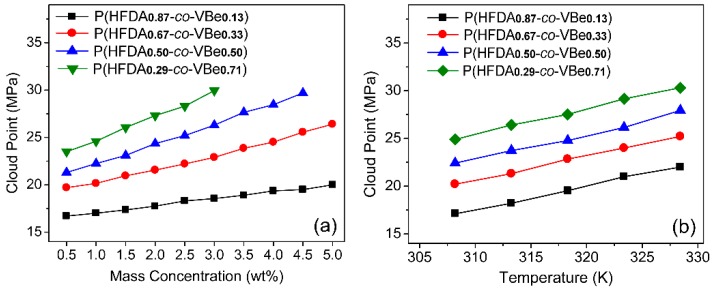
Cloud point pressures of the co-polymer–CO_2_ solutions versus mass concentration at 308.2 K (**a**) and temperature (**b**) at 1 wt. % of concentration.

The cloud point pressure of the co-polymer–CO_2_ solution with mass concentration at 308.2 K and temperature at 1 wt. % of concentration are shown in Figure 4. As observed in Figure 4a, the cloud point pressures of the co-polymers gradually enhanced with an increase in the mass concentration. The cloud point curves also reflected the influence of the VBe molar ratio on cloud point pressures. With an increase in the molar ratio of VBe, the cloud point pressures of the co-polymers increased rapidly. It seemed that the VBe content had greater effect than the mass concentration in determining the solubility of the co-polymers. The cloud point pressure of the co-polymers increased with an increase in temperature, which is shown in Figure 4b. With the temperature rose from 308.2 K to 328.2 K, the cloud point pressures of the four co-polymers were found to increase by about 5 MPa equivalently. Obviously, the temperature has a significant effect on the solubility of the co-polymers in SC-CO_2_. This may be related to the change of CO_2_ properties caused by the change in temperature. When the temperature rose, the density and dielectric constant of CO_2_ decreased, so its dissolubility decreased [52]. To counteract the downfall of CO_2_ dissolubility, the pressure must be increased to enhance the co-polymer–CO_2_ interactions, which helped to balance the intermolecular interactions in the solutions [53].

The radial distribution function (RDF) is a common function used to study the microstructures and evaluate the intermolecular interactions, which refers to the spherically symmetric distribution probability of other particles in a space centered on a given particle. This can be calculated as follows [54,55]:

(3) $$g_{\alpha \beta }(r)=\frac{V}{N_{\alpha }N_{\beta }}\langle \sum_{i=1}^{N_{\alpha }}\frac{n_{i\beta }(r)}{4\pi r^{2}\Delta r}\rangle ,$$

where *g_α__β_*(*r*) is the value of radial distribution function, *N_α_* is the number of *α* particles; N*_β_* is the number of *β* particle; *V* is the volume of box simulated; and *n_iβ_*(*r*) is the number of *β* particles in the space of (*r*, *r +* Δ*r*) centered on *α* particles.

**Figure 5:**
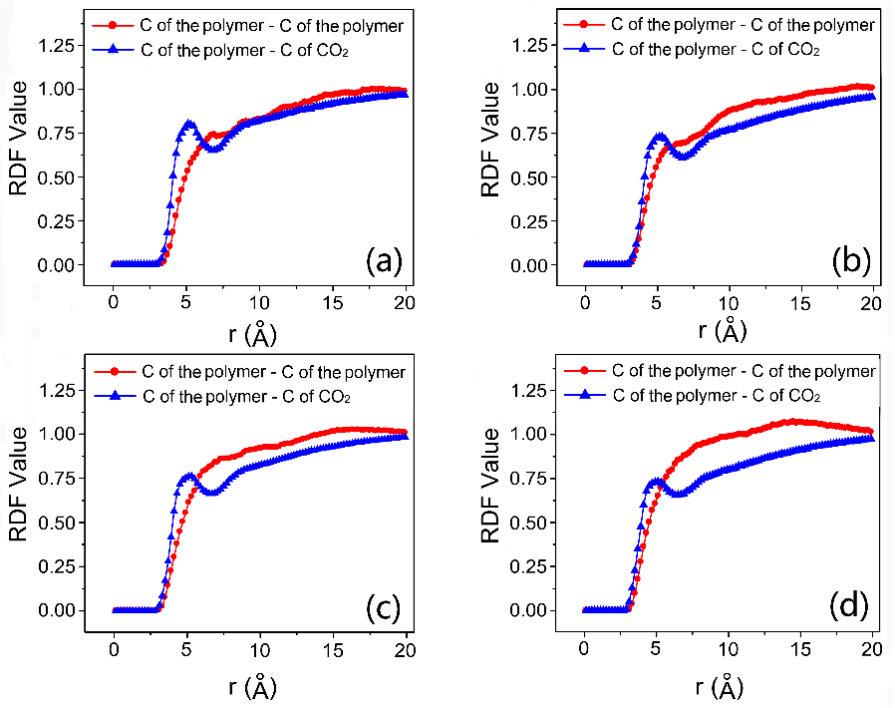
Intermolecular RDFs of the carbon-carbon pairs of polymer–polymer and polymer–CO_2_. (**a**) P(HFDA_0.87_-*co*-VBe_0.13_); (**b**) P(HFDA_0.67_-*co*-VBe_0.33_); (**c**) P(HFDA_0.50_-*co*-VBe_0.50_); (**d**) P(HFDA_0.29_-*co*-VBe_0.71_).

Solvation plays an important role in controlling the solubility of polymer in SC-CO_2_ [56]. The solvation behavior of the polymer in SC-CO_2_ depends on the relative strength of the solute-solvent interaction to the solvent-solvent interaction [57]. The enrichment or dispersion of CO_2_ around the polymer would determine the tendency of the polymer to gather or disperse in CO_2_ [58]. Intermolecular RDF has been used to evaluate the miscibility of polymer–solvent systems by investigating the solvation behavior. If the RDF values of atom pairs between polymer and solvent are greater than or almost equal to those of polymers, indicating the richness of the solvent around the polymer, the polymer would dissolve easily and may exhibit good solubility in the solvent [19,59,60]. The RDF statistical results for the C of the polymer–C of the polymer and C of the polymer–C of CO_2_ are shown in Figure 5. For the system of P(HFDA_0.87_-*co*-VBe_0.13_)-CO_2_, the RDF curve of C of the polymer–C of CO_2_ was almost equal to that of C of the polymer–C of the polymer as observed in Figure 5a. For the other three co-polymers, the RDF curves of C of the polymer–C of CO_2_ became significantly lower than that of C of the polymer–C of the polymer as shown in Figure 5b–d. With an increase in VBe content, the curve gaps between C of the polymer–C of CO_2_ and C of the polymer–C of the polymer increased gradually. The RDF results showed that the co-polymers with lower VBe content were more likely to be surrounded and solvated by CO_2_, thus indicating that they were more soluble in CO_2_. 

Generally speaking, the solubility of polymers in SC-CO_2_ is dominated by polymer–CO_2_ interactions, polymer–polymer interaction and chain flexibility. The CO_2_-soluble polymers have strong polymer–CO_2_ interaction and weak polymer–polymer interactions, which is reflected by low surface tension. In addition, the high chain flexibility results in the polymers exhibiting the high entropy of mixing in CO_2_, which is reflected by the low glass transition temperature (*T_g_*). The experimental results in Table 2 showed that with an increase of the molar ratio of VBe, the surface tension and *T_g_* increased gradually, which indicated that the enhancement of polymer–polymer interaction and the decrease of chain flexibility. The polymer–CO_2_ interaction strength could be characterized by the interaction energy quantitatively, with a greater absolute value of the interaction energy indicating a stronger polymer–CO_2_ interaction. The interaction energies of co-polymer–CO_2_ and PHFDA–CO_2_ were examined by MD. As shown in Table 3, the interaction energy of PHFDA–CO_2_ was significantly greater than those of the four co-polymers. The interaction energy of co-polymer–CO_2_ trended downward along with the VBe molar ratio. This indicated that the import of VBe weakened the co-polymer–CO_2_ interaction.

In summary, with an increase in VBe content, the co-polymer–CO_2_ interaction energy decreased, the polymer–polymer interaction enhanced, the chain flexibility declined and resulted in unfavorable entropy of mixing, which resulted in a decrease the co-polymer solubility and an increase in the cloud point pressure of the co-polymer–CO_2_ system.

### 3.3. Thickening Capability of Co-Polymers in SC-CO_2_

**Figure 6:**
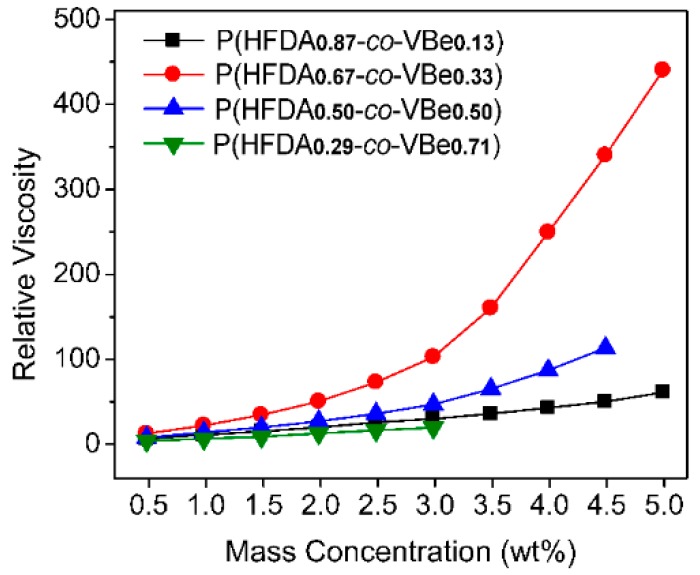
Relative viscosities of the co-polymer–CO_2_ systems at 308.2 K and 30 MPa versus mass concentration.

The variations of the relative viscosity of the co-polymer–CO_2_ solutions against mass concentration at 308.2 K and 30 MPa were used to evaluate the viscosification, which is shown in Figure 6. This indicated that all the co-polymers could thicken SC-CO_2_ and the thickening capability increased with greater mass concentration. Unlike the solubility, the thickening capability did not vary with the VBe molar ratio linearly with a maximum occurring at 33.0 mol %. The P(HFDA_0.67_-*co*-VBe_0.33_) could enhance the viscosity of SC-CO_2_ by 438 times at 5 wt. %. The P(HFDA_0.87_-*co*-VBe_0.13_) with lower content of VBe did not enhance the viscosity of SC-CO_2_ significantly, while the thickening capabilities of P(HFDA_0.29_-*co*-VBe_0.71_) and P(HFDA_0.50_-*co*-VBe_0.50_) with higher content of VBe were also limited.

**Figure 7:**
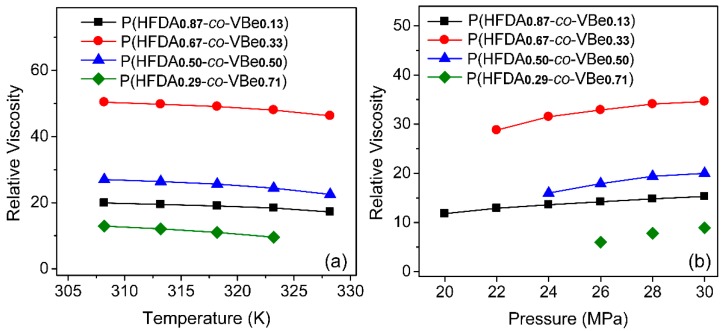
Relative viscosities of the co-polymer–CO_2_ systems versus temperature (**a**) and pressure (**b**).

Figure 7a,b showed the relative viscosity of co-polymer–CO_2_ solution with 2 wt. % of concentration versus temperature at 30 MPa and versus pressure at 35 °C, respectively. As shown in the figure, the relative viscosity diminished with an increase in temperature, although this was enhanced with an increase in pressure. With an increase in temperature, the dissolubility of CO_2_ decreased, the co-polymer chains crimped, and the size of hydrodynamics became smaller, so the degree of intermolecular association decreased, leading to a decrease in thickening capability. At higher pressures, the solubility parameter and dissolubility of CO_2_ was enhanced [52,53], and the co-polymer chains were more stretched in solution, with the larger hydrodynamic size allowing the generation of stronger thickening capability through more intermolecular association.

**Figure 8:**
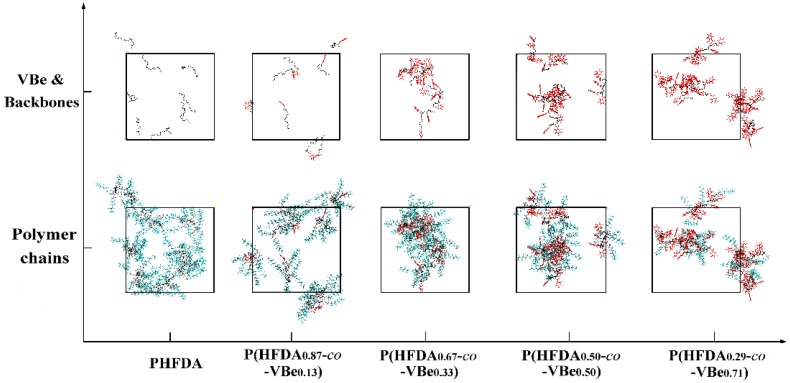
Equilibrium conformations of five CO_2_–polymer systems at 308.2 K and 25 MPa. All the CO_2_ molecules were deleted to highlight the polymer chains (**lower**) and the specific functional groups and backbones (**upper**).

To understand the thickening capability of the co-polymers intuitively, the equilibrium conformations of the co-polymers and the PHFDA in SC-CO_2_ obtained by molecular dynamics simulation were analyzed. As shown in Figure 8, there was no effective aggregation between the polymer chains for the PHFDA. The co-polymers containing VBe groups exhibited aggregation between polymer chains in different levels. It may be inferred that PHFDA lacked the basic power of triggering intermolecular association due to the weak polymer–polymer interaction, while the VBe group in the co-polymers could provide the driving power. The most obvious intermolecular association was initiated by P(HFDA_0.67_-*co*-VBe_0.33_), which formed effective molecular aggregates. The P(HFDA_0.87_-*co*-VBe_0.13_) with lower VBe content exhibited less inter-chain aggregation compared to P(HFDA_0.67_-*co*-VBe_0.33_). It was inferred that its poor thickening capabilities rooted in the lack intermolecular aggregation was due to the shortage of VBe groups. However, the excess of VBe groups would generate more intramolecular π-π stacking and a decrease in intermolecular π-π stacking, just as the equilibrium conformations shown in Figure 8. Therefore, the thickening capabilities of P(HFDA_0.50_-*co*-VBe_0.50_) and P(HFDA_0.29_-*co*-VBe_0.71_) with higher VBe content were not as good as P(HFDA_0.67_-*co*-VBe_0.33_). In this work, 33.0 mol % of VBe content of the co-polymer was the optimum value for generating the strongest intermolecular association and thus the best thickening performance. 

**Figure 9:**
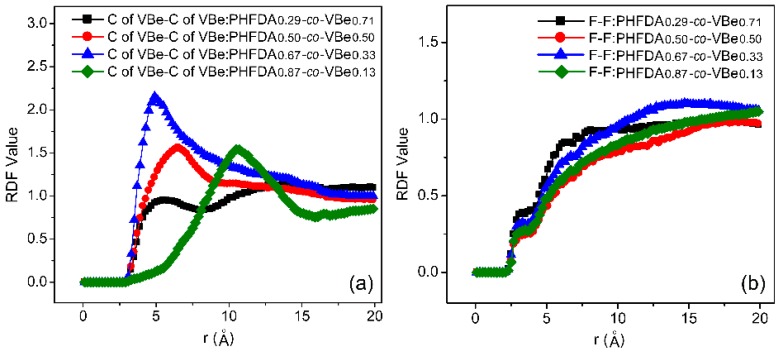
Radial distribution functions of the intermolecular C-C pairs of VBe groups (**a**) and F-F pairs of HFDA groups (**b**).

The peak position and peak value of the radial distribution function could indicate the strength of the inter-group interaction indirectly. A smaller peak position and higher peak value represented the stronger interaction strength. Intermolecular RDFs for C-C pairs of the VBe groups and F-F pairs of the HFDA groups were used to further investigate the microstructure of the four co-polymer–CO_2_ systems quantitatively, with the results shown in Figure 9. As shown in Figure 9a, the distance of the RDF curves that achieved the values of greater than zero was about 2.9 Å. This indicated that the intermolecular interactions of VBe groups in the co-polymers were dominated by van der Waals forces. The RDF values and positions of the peaks were significantly different for the four polymer–CO_2_ systems. The RDF curve peak of P(HFDA_0.67_-*co*-VBe_0.33_) was the highest, while a peak appeared at the distance of 4.9 Å. The RDF curve peak heights of P(HFDA_0.87_-*co*-VBe_0.13_) and P(HFDA_0.50_-*co*-VBe_0.50_) were almost the same, but the peak of the former appeared at 10.7 Å, which was farther away than the 6.5 Å of the latter. No peak could be observed in the RDF curve of P(HFDA_0.29_-*co*-VBe_0.71_), which had the smallest RDF value. As shown in Figure 9b, all the curves showed similar trend and no obvious peak was observed. This indicated that the intermolecular interaction between HFDA groups in co-polymer was not strong enough to generate effective intermolecular associations. It may be related to the strong electronegativity and fluorine repulsion of F atoms in the HFDA group. By comparing the data in Figure 5 and Figure 9, it is not difficult to find that intermolecular aggregation occurs between the VBe groups not between HFDA groups. According to the results of experiments and simulations, the thickening capabilities of the co-polymers in SC-CO_2_ should be attributed to the π-π stacking of VBe group.

## 4. Conclusions

Four P(HFDA-*co*-VBe) co-polymers with different compositions were synthesized and evaluated. The molecular models of co-polymer–CO_2_ systems were established to study the molecular behaviors and intermolecular interactions by the all-atom MD simulation. We found that the π-π stacking of the VBe group played a decisive role in thickening SC-CO_2_. The solubility of the co-polymers decreased with an increase in VBe content, while the thickening capability did not vary with the VBe molar ratio linearly. The relative viscosity of the co-polymers reached its maximum of 438 at a VBe molar ratio of 33.0% in this work. Less VBe content resulted in the lack of intermolecular π-π stacking, while excessive VBe content generated more intramolecular π-π stacking and less intermolecular π-π stacking. This work provided the molecular insights into the relationship of co-polymer structure with the activity by the study of intermolecular interactions. The thickening capability of ternary co-polymer or polymer surfactant in SC-CO_2_ will be discussed in future studies.

## Figures and Tables

**Table 1:** The systems simulated by all-atom molecular dynamics.

Run	System	*M_n_* of One Chain	Number of Chains	Number of HFDA Units in One Chain	Number of VBe Units in One Chain	Number of CO_2_
1	CO_2_+PHFDA	3630	8	7	0	2000
2	CO_2_+P(HFDA_0.87_-*co*-VBe_0.13_)	3777	8	7	1	2000
3	CO_2_+P(HFDA_0.67_-*co*-VBe_0.33_)	3555	8	6	3	2000
4	CO_2_+P(HFDA_0.50_-*co*-VBe_0.50_)	3333	8	5	5	2000
5	CO_2_+P(HFDA_0.29_-*co*-VBe_0.71_)	3556	8	4	10	2000
6	PHFDA	3630	8	7	0	0
7	P(HFDA_0.87_-*co*-VBe_0.13_)	3777	8	7	1	0
8	P(HFDA_0.67_-*co*-VBe_0.33_)	3555	8	6	3	0
9	P(HFDA_0.50_-*co*-VBe_0.50_)	3333	8	5	5	0
10	P(HFDA_0.29_-*co*-VBe_0.71_)	3556	8	4	10	0

**Table 2:** Experimental results for the synthesis and physical properties of the co-polymers.

Run	Sample	*M_w_* (g/mol) ^a^	Composition ^b^	Feed Ratio	*T_g_* (°C) ^c^	γ (mN/m^−1^) ^d^
			HFDA:VBe	HFDA:VBe		
1	P(HFDA_0.87_-*co*-VBe_0.13_)	152,100	87:33	85:15	52.6	27.4
2	P(HFDA_0.67_-*co*-VBe_0.33_)	145,600	67:33	70:30	55.4	29.7
3	P(HFDA_0.50_-*co*-VBe_0.50_)	142,900	50:50	50:50	58.1	32.5
4	P(HFDA_0.29_-*co*-VBe_0.71_)	150,400	29:71	30:70	62.8	35.8

^a^ Measured with Wyatt DAWN HELEOS Multi-Angle Static Light Scattering Scatter; ^b^ Determined by ^1^H-NMR using Bruker-400 MHz NMR; ^c^ Measured by Mettler-Toledo Differential Scanning Calorimeter; ^d^ Determined by contact angle measurements of water-polymer and hexadecane-polymer with Krishna DSAHT Contact Angle Meter.

**Table 3:** Absolute value of the interaction energy between polymer chains and CO_2_ at 308.2 K and 25 MPa (energy unit: kJ/mol).

Run	System	*E_chain−CO_*_2_	*E_chain_*	*E_CO_*_2_	*E_inter_*
1	CO_2_+PHFDA	4341.63	1357.22	1556.01	1428.40
2	CO_2_+P(HFDA_0.87_-*co*-VBe_0.13_)	3986.58	1089.34	1698.63	1198.61
3	CO_2_+P(HFDA_0.67_-*co*-VBe_0.33_)	3643.29	773.12	1805.42	1064.75
4	CO_2_+P(HFDA_0.50_-*co*-VBe_0.50_)	3327.36	680.63	1718.76	927.98
5	CO_2_+P(HFDA_0.29_-*co*-VBe_0.71_)	3438.42	769.19	1960.29	708.4
